# Fertility Workup With Video Consultation During the COVID-19 Pandemic: Pilot Quantitative and Qualitative Study

**DOI:** 10.2196/32000

**Published:** 2022-02-07

**Authors:** Hilde Grens, Jan Peter de Bruin, Aleida Huppelschoten, Jan A M Kremer

**Affiliations:** 1 Department of Obstetrics and Gynaecology Jeroen Bosch Hospital 's-Hertogenbosch Netherlands; 2 Department of Obstetrics and Gynaecology Catharina Hospital Eindhoven Netherlands; 3 Scientific Institute for Quality in Healthcare Radboud University Medical Centre Nijmegen Netherlands

**Keywords:** COVID-19, patient centeredness, video consultation, fertility care, telemedicine, shared decision making

## Abstract

**Background:**

Due to the COVID-19 pandemic, major parts of elective health care in the Netherlands, such as reproductive medicine, were paused. When health care was resumed, video consultation was used as a new solution to continue consultations with the new governmental rules of social distancing. Prior to this COVID-19 situation, video consultation was not used extensively in the Netherlands; therefore, physicians and patients are not familiar with this way of consultation.

**Objective:**

The purpose of this study was to measure the level of patient centeredness and shared decision making in infertile couples who have undergone fertility workup through video consultation.

**Methods:**

This is a questionnaire study with an additional qualitative part for a more in depth understanding. Infertile couples (ie, male and female partners with an unfulfilled wish for a child after 1 year of unprotected intercourse) were referred to a fertility center and underwent fertility workup through video consultation. The fertility workup consisted of 2 separate video consultations, with diagnostic tests according to a protocol. After the last video consultation couples received a digital questionnaire, which consisted of a modified version of the Patient-Centered Questionnaire-Infertility (PCQ-I) and CollaboRATE questionnaire. Fifty-three eligible infertile couples were approached, and of these, 22 participated. Four women were approached for a semistructured interview.

**Results:**

The median score on the modified PCQ-I (scale of 0 to 3) was 2.64. The highest rating was for the subscale communication and information, and the lowest rating was for the subscale organization of care. The median score on the CollaboRATE questionnaire (scale of 1 to 9) was 8 for all 3 subquestions. Patients mentioned privacy, less travel time, and easy use of the program as possible benefits of video consultation. However, patients preferred the first consultation with their physician to be face-to-face consultation as video consultation was considered less personal.

**Conclusions:**

The high levels of patient centeredness and shared decision making show that video consultation is a promising way of providing care remotely, although attention has to be payed to mitigate the more impersonal setting of video consultation when compared with face-to-face consultation.

## Introduction

In the initial months of 2020, the COVID-19 pandemic put a stop to most societal activities and major parts of elective health care, such as reproductive medicine. If possible, on-going in vitro fertilization cycles were completed, after which fertility treatments were postponed, based on the advice of both the Dutch and European scientific societies. When elective health care was restarted after a few months, medical departments faced new challenges, having to implement governmental rules on social distancing and minimizing patient traffic in hospitals. One of the solutions for these challenges, in order to continue consultation without physical visits to the hospital, is video consultation.

Video consultation has existed for some time and was mentioned before as a solution to scarcity in health care. Although it was not being used extensively in the Netherlands, it seems to have advantages for patients, such as less travel time and parking costs, and avoidance of waiting in the hospital. Besides, patients are not dependent on others for hospital visits and can experience privacy in the comfort of their own homes. However, technical issues, inferior computer skills, data security issues, and a less personal approach compared with face-to-face consultation are possible pitfalls [1,2]. The less personal approach might be the reason that video consultation has rarely been used during initial consultations. This is supported by the results of a survey by the Dutch Patient Federation and the Amsterdam UMC. Only 6.7% of the respondents opted for video consultation during first contact with a doctor [3].

We have been applying video consulting for first consultations for several years in our project *Fertility Consult*. It involves a secured online platform [4] for infertile couples who are seeking independent advice on fertility treatment. The service includes online questionnaires about prior fertility treatments and medical history, followed by video consultation with a fertility expert. Despite the fact that the video consultation was the first contact couples had with the physician, they were positive about the service. The first results on the patient centeredness of *Fertility Consult* have recently been published [5].

The decision to pause elective health care during the COVID-19 pandemic had a big impact on all patients, with infertility patients being no exception to the rule. It is known that involuntary childlessness is a heavy burden on patients’ quality of life. Therefore, infertile patients benefited from quick resumption of care, with a form of care that fitted with the social distancing rules (ie, video consultation). To assess how this care was experienced, a pilot study was set up at the Reproductive Medicine Centre in Jeroen Bosch Hospital, The Netherlands. The aim of this pilot study was to explore patient experiences with a fertility workup through video consultation and extract the possible advantages and disadvantages of video consultation. These results can be used to improve the implementation and quality of video consultation in daily fertility care.

## Methods

### Overview

We performed a study consisting of both quantitative and qualitative parts to evaluate the experiences of infertile couples with an online fertility workup and obtain more in-depth understanding of their experiences.

### Study Design and Recruitment

For the online fertility workup, couples with an unfulfilled wish for a child were referred by their general practitioner after having unprotected intercourse for more than 12 months, just as before the COVID-19 pandemic. Couples completed a questionnaire about their medical situation, after which a video consultation was scheduled instead of a regular appointment in the outpatient clinic. This video consultation was carried out by either a gynecologist or a fertility physician. The program used for the video consultation was called Webcam Consult [6], which works according to the international quality standard ISO 27001 and is linked to the local electronic hospital patient file. Based on the video consultation, the woman was invited to the hospital for a gynecological ultrasound. At this appointment, the woman received laboratory forms for her and her partner for blood and sperm analysis, if necessary.

Consequently, a second video consultation was performed, with the same physician as in the first video consultation, to discuss the results, diagnosis, and possible therapy. After the second video consultation, all couples were asked to participate in the study, which was performed between May and June 2020. Those who did not understand the Dutch language were excluded, as the questionnaire was only available in Dutch. One questionnaire was sent per couple, which could be completed in a secured online environment. Nonresponders received 2 reminders. Four patients were approached, at random, to participate in a semistructured interview. A separate written consent form was collected before the interview.

As the study had minimal impact on the study subjects (ie, filling in a single questionnaire or participating in a single semistructured interview), it was judged that the Dutch Medical Research with Human Subjects Law was not applicable. Therefore, approval of the medical ethics committee was not needed.

### Quantitative Part

The quantitative part consisted of a modified version of the validated Patient-Centered Questionnaire-Infertility (PCQ-I) [7] and the CollaboRATE questionnaire [8] measuring patient centeredness and shared decision making, respectively.

The original PCQ-I consists of 7 subscales with 46 questions covering many different subjects of patient-centered infertility care, such as information provision, communication, and continuity of care. The PCQ-I scale has a range from 0 to 3, where a higher score implies a higher level of patient centeredness. Since not all questions were suitable for the specific video consultation setting, we used the modified PCQ-I, as used in the study by Huppelschoten et al [5], which consists of 18 questions. The main modifications were done in the following categories: accessibility, information, and cooperation within care, since a number of aspects in these questions are not applicable for a setting with a video consultation [5].

The CollaboRATE questionnaire consists of the following 3 questions: How much effort was made to help you understand your health issue? How much effort was made to listen to the things that matter most to you about your health issues? and How much effort was made to include what matters most to you in choosing what to do next? Patients answer these questions on a 10-point Likert scale from 0 (“no effort was made”) to 9 (“every effort was made”).

In addition to the 2 questionnaires, some questions were added about the technical and practical aspects of video consultation.

### Qualitative Part

For the qualitative part, several women were approached for semistructured interviews to investigate in more depth the possible advantages and disadvantages of participating in video consultation. A topic list was drafted in advance, based on available literature, and discussed with a small team of experts.

The interviews were held by telephone. The conversation was recorded and transcribed to extract patient comments. Interviews were held until saturation of information was reached. The participants of the interviews also completed the PCQ-I and CollaboRATE questionnaire.

### Data Storage and Privacy

The data from the questionnaires were collected from a secure online environment called “Digitale kinderwens poli” (Digital Childwish clinic), which is used in a clinical setting, and exported to Excel. The data from the questionnaire and from the semistructured interviews were handled by a local investigator, who had access to the participant code. For the other members of the research group, the data were anonymous. The data were stored according to the standards of the local facility.

### Statistical Analysis

The demographics and background characteristics of the participants and the results of the PCQ-I and CollaboRATE questionnaire were analyzed using descriptive statistics. The normally distributed outcome measures are represented by means of the means with standard deviations, and the nonnormally distributed outcomes are represented by means of the medians. Analyses were performed using SPSS version 22 (IBM Corp).

## Results

### Quantitative Part

Out of the 53 couples who completed the online fertility workup, 22 responded (response rate 42%). The mean age was 31.7 years (SD 4.1 years). The characteristics of all patients are summarized in Table 1.

**Table 1:** Baseline characteristics of the participants (N=22).

Characteristic		Value
Age (years), mean (SD)		31.7 (4.1)
**Level of education^a^, n (%)**		
	High	14 (64)
	Other	8 (36)
**Ethnic background, n (%)**		
	Dutch	19 (85)
	European	1 (5)
	Asian	1 (5)
	Other	1 (5)
**Duration of infertility, n (%)**		
	Less than 2 years	19 (87)
	2 to 5 years	3 (13)
Pregnant during the completion of the questionnaire, n (%)		2 (9)

^a^High education indicates higher professional education or university education.

The results of the PCQ-I and CollaboRATE questionnaire are presented in Table 2. The median of the total PCQ-I score was 2.64 (range 0-3). The highest rating was for the subscale communication and information (median 2.83), and the lowest rating was for the subscale organization of care (median 2.33). For the CollaboRATE questionnaire, the median score for all 3 questions was 8 on a scale of 1 to 9.

**Table 2:** Questionnaire results (N=22).

Outcome values		Value
**PCQ-I^a^ questionnaire (range 0-3), median (range)**		
	Total score	2.64 (1.78-2.94)
	Communication and information score	2.83 (2.00-3.00)
	Respect for patients’ values score	2.75 (0.50-3.00)
	Staff’s competence score	2.70 (1.80-3.00)
	Organization of care score	2.33 (1.33-3.00)
**CollaboRATE questionnaire (range 0-9),** **m** **edian (range)**		
	Helping to understand health issues score	8 (2-10)
	Listen to things that matter most score	8 (5-10)
	What to do next score	8 (5-10)
**Personal experience**		
	Overall satisfaction with the Centre for Reproductive Medicine (range 0-10), median (range)	8 (5-10)
	Technical problems, n (%)	9 (40)

^a^PCQ-I: Patient-Centered Questionnaire-Infertility.

### Qualitative Part

Four interviews were held with female patients. The interviews were analyzed, and quotations were categorized. Acceptance of video consultation was high, given the current COVID-19 situation. Three women expressed that for the first consultation, in a more optimal situation without the COVID-19 pandemic, they would prefer to visit their physician in the hospital.

Example quotations can be found in Textbox 1. Most reported benefits were shorter travel time, a secure feeling concerning privacy, and easy use of the video consultation program.

**Quotations from interviews (N=4).**

**Information**

- A video consultation is a good replacement for a conversation, a telephonic call is inferior.

- It worked perfect to hear the results of the tests per video consultation.

**Privacy**

- I was not worried about the privacy.

- I felt very secure in my own environment.

**Communication/respect for patient value**

- I would prefer to talk to my physician face-to-face on the first appointment, because it is possible that video consultation lacks body language.

- I found the video consultation a bit impersonal.

**Involvement of partner**

- I could evaluate the appointment with my partner immediately, because we were already home.

**Organization of care**

- I don’t like waiting in waiting rooms, and now I was able to wait at home.

- We saved a lot of time, without traveling to the hospital.

**Technical aspect**

- Everything went very smooth, no problems.

- There was a clear instruction beforehand.

- On beforehand I was a bit worried if the connection would be good enough.

- We didn’t have any sound, so the doctor called us. That made it a bit chaotic.

## Discussion

### Principal Findings

This is one of the first studies to explore patient experiences with video consultation in the context of the first consultation. In general, patients perceived a high level of patient centeredness as reflected in a high score on the PCQ-I. Video consultation also scored high in shared decision making on the CollaboRATE questionnaire. During the interviews, patients expressed a relatively high satisfaction rate with video consultation, although they did express that their preference would be to meet their physician face-to-face for the first consultation.

### Strengths and Limitations

An important strength of this study is that, to our knowledge, this is one of the first studies to explore patient experiences in their first contact with a new medical department. As both quantitative and qualitative techniques were used, we were able to gather more in-depth information about patient experiences. Besides, we used 2 validated questionnaires, which can be used in the future to compare results.

Some limitations should be mentioned as well. First, since this was a pilot study, the sample size was rather small. The response rate was only 42%, and it would be interesting to know why the other patients did not respond. Second, the relatively high median scores on the PCQ-I and CollaboRATE questionnaire may be affected by the circumstances of the current COVID-19 pandemic [9]. The patients who participated in our study were the first patients to receive fertility consultation after all fertility treatments and appointments had been paused. Therefore, there might have been bias, as the patients were probably relieved to be able to start fertility workup.

### Comparison With the Literature

In general, literature on video consultation in health care is limited. Often, only small groups were studied in different settings and almost exclusively using video consultation in the context of a follow-up consultation. Previous Dutch research did study the preferences of a larger group (968 patients), but only 1.7% of the responders had prior experience with video consultation [3]. In another systematic review, physicians seemed to prefer face-to-face consultation, but patients were just as satisfied with video consultation as with face-to-face consultation and preferred it to telephone and email consultations [10].

A British study in primary care showed that video consultation involved less information when compared with face-to-face contact. For example, during video consultation, patients raised fewer issues, the physician provided less information, and the psychosocial context was more often lost [11]. Our study had similar results; in some but not in all cases, patients felt the possibility to express their emotions during the video consultation.

In addition to the content of the video consultation, its implementation is important. In this pilot study, technical problems were recorded in 40% of cases, which is a relatively high percentage. A different British study emphasized this. In the study, clinicians and patients were interviewed after a video consult, and both groups mentioned that technological problems sometimes disrupted the consultation [12]. Both the loss of possible content and the need of higher technical skills entail risks, especially in those patients who already have a psychosocial or socioeconomic disadvantage. To overcome these challenges, the practicing physician must be aware of the possible pitfalls of video consultation and act accordingly. For example, by actively asking patients about the emotional consequences of involuntary childlessness, they are invited to express their emotions.

### Conclusions

This pilot study explored infertile patients’ experiences using video consultation for their first contact with a gynecologist, after being referred for an unfulfilled wish of having a child. This study showed high scores for patient centeredness and shared decision making. Video consultation is a way of providing care remotely and could potentially be superior to other forms of telecommunication, such as telephone and email contact. Although the acceptance of video consultation might be influenced by COVID-19 circumstances, we do think that the results are promising for the future.

As a next step, we will continue research on video consultation in fertility care. A randomized controlled trial will compare online fertility workup with the standard fertility workup involving face-to-face consults (Dutch trial register number 8554) in terms of patient centeredness and health care costs. Future studies should also focus on how to promote the implementation of video consultation in our current health care setting and minimize technical issues, so that video consultation will be continued after the COVID-19 pandemic.

## Abbreviations

PCQ-I: Patient-Centered Questionnaire-Infertility
